# A Perennial Threat: A Case Series of Tree-Related Neurotrauma

**DOI:** 10.7759/cureus.17104

**Published:** 2021-08-11

**Authors:** Cody Reiber, Alan A Stein, Ivan Miller, Omar Tarawneh, Syed Faraz Kazim, Meic H Schmidt, Chad D Cole, Christian A Bowers

**Affiliations:** 1 School of Medicine, New York Medical College, Westchester, USA; 2 Neurosurgery, New York Medical College, Westchester, USA; 3 Emergency Medicine, Westchester Medical Center, Valhalla, USA; 4 Neurosurgery, University of New Mexico Hospital, Albuquerque, USA; 5 Neurosurgery, University of New Mexico School of Medicine, Albuquerque, USA

**Keywords:** tree collisions, neurotrauma, outcomes, injury patterns, accidents

## Abstract

Background

Despite their devastating nature, injuries due to tree-related- traumas are sparsely reported in the literature. Over the last several years, the incidence of tree-related traumatic injuries presenting to our level one trauma center, in Westchester, New York, has been concerning. The present study was undertaken to evaluate the clinical presentation, injury pattern, and outcomes of tree-related neurotrauma at our institution. In addition, we describe the injury pattern and medical management of several relevant cases of tree-related neurotrauma.

Methods

We conducted a retrospective analysis of tree-related neurotrauma over a five-year period from January of 2014 to March of 2019 at Westchester Medical Center (WMC) and Maria Fareri Children’s Hospital, a level one trauma center. Patients presenting with neurotrauma that necessitated neurosurgical care were eligible for inclusion in this case series. Tree-related injury was defined as any trauma that was sustained as a direct result of collision with a tree.

Results

We identified 21 patients who sustained tree-related trauma. The cohort age ranged from 15 to 68 (mean=38 years). Injuries included seven skull fractures, four cases of subdural hematoma (SDH), six cases of intracranial hemorrhagic contusion, 14 spinal fractures, three cases of epidural hematoma (EDH), one case of spinal cord contusion, three vascular injuries, one case of dural laceration, and one case of pneumocephalus, with several patients suffering multiple injuries. Of the 21 patients, seven were female, and 12 were injured when their motor vehicle struck a tree. All but four patients were taken to the operating room for neurosurgical treatment, and nine of 21 patients were taken emergently to the operating room upon arrival.

Conclusion

The potential for serious head injuries with long-term neurologic sequelae exists with tree-related trauma. Clinicians should be advised of the challenging management of injuries secondary to tree-related trauma, and a greater emphasis should be placed on raising awareness of these accidental, but devastating injuries. Finally, a great majority of these injuries can be prevented or reduced in severity through helmet use and by adhering to safety guidelines.

## Introduction

The Northeast of the United States has the highest concentration of forested areas in the country. The statewide tree cover of the state of New York is 65.0 percent by land area (roughly 20.38 million acres), which is considerably higher than the average percent tree cover of the continental United States as a whole (34.2%) [1]. Since 2014, the incidence of tree-related traumatic injuries presenting to the emergency department at our level one trauma center, in Westchester, New York has been alarming. Those requiring emergent neurosurgical services are particularly challenging as even with timely treatment, patients may experience long term-neurological deficits due to their injuries.

The available literature regarding this subject shows a paucity of data describing tree-related neurotrauma, with the majority of studies limited to injuries sustained treehouse and tree-stand fall [2]. Despite the relatively high frequency and severity of these injuries, tree-related trauma has received little attention in the neurosurgical literature. Walsh et al. published a study assessing the prevalence and outcome of injuries related to falling trees and tree branches, however, the authors did not elaborate on the inpatient course or prognosis of these patients [3]. Moreover, a case series of tree-related trauma published by Hakakian et al. addressed all forms of tree-related trauma and did not focus specifically on injury to the brain and spinal cord [4].

Our institution has recently treated multiple patients with severe neurological injuries related to tree-related trauma, thus prompting an analysis of the nature and mechanism of these injuries. For the purpose of this study, we defined tree-related injury as injury caused by falling trees, patients falling out of trees, and motor vehicle accidents involving collisions with trees. Like other forms of blunt or penetrating trauma, tree trauma can cause significant neurological injury, necessitating emergent neurosurgical intervention. The relative abundance of trees in the area increases the risk of their involvement in trauma and necessitates the reporting of the injuries sustained and the treatment modalities employed. In this study, we present a series of 21 patients with tree-related neurotrauma treated emergently at our institution from 2014 to 2019. Additionally, we describe the patterns of injuries sustained, procedures performed, and patient outcomes.

## Materials and methods

We retrospectively reviewed the Westchester Medical Center (WMC) and Maria Fareri Children’s Hospital Emergency Department Database to analyze tree-related neurotrauma over a five-year period (January 2014 - March 2019). IRB approval was obtained from Westchester Medical Center (IRB protocol # 14085). The WMC/Maria Fareri Children’s Hospital is a tertiary medical center serving hundreds of thousands of patients in Westchester County, New York, and an American College of Surgeons-accredited Level one Adult and Pediatric Trauma Center. Inclusion criteria included patients presenting with neurotrauma following tree-related trauma that necessitated neurosurgical intervention. Tree-related injury was defined as any trauma that was sustained as a direct result of a collision with a tree. Patient demographics, imaging, surgical treatment, complications, and long-term outcomes were recorded. Imaging of patients was taken during and after treatment, and patients’ consent was obtained for publication of the imaging.

## Results

A total of 21 patients (seven females and 14 males) ages 15 to 68 (mean age 38.0) were eligible for study inclusion(Table 1). Injuries sustained included seven skull fractures, four cases of subdural hematoma (SDH), six cases of intracranial hemorrhagic contusion, 14 spinal fractures, three cases of epidural hematoma (EDH), one case of spinal cord contusion, three vascular injuries, one case of dural laceration, and one case of pneumocephalus, with some patients suffering multiple injuries. Of the 21 patients, 12 sustained injuries when their motor vehicle collided with a tree. All but four patients were taken to the operating room for neurosurgery. The four patients who did not require neurosurgical intervention were admitted to the intensive care unit. Finally, nine of 21 patients evaluated were treated emergently in the operating room upon arrival.

**Table 1:** Clinical characteristics, neuroimaging findings, neurosurgical intervention, and outcomes of 21 patients with tree-related neurotrauma in our study cohort. ACDF: anterior cervical discectomy and fusion, C: cervical, CSF: cerebrospinal fluid, EDH: epidural hematoma, EVD: extraventricular drain, F: female, fx: fracture, ICA: internal carotid artery, L: lumbar, M: male, n/a: not applicable, POD: postoperative day, T: thoracic, TBI: traumatic brain injury

Case #	Age (yrs)	Sex	Mechanism of injury	Imaging finding	Neurosurgical intervention	Outcome	Complication(s)
1	26	M	Motorcycle into a tree while intoxicated	SDH, C5-7 fxs, EDH of the spinal cord with compression	C6 corpectomy with fusion of C5-C7, C6-C7 laminectomy with C4-T2 fusion	Discharge to TBI and SCI rehabilitation	n/a
2	42	M	Jumped out of a tree in a suicide attempt while intoxicated	L2 burst fx	T12-L4 laminectomy and fusion, and decompression of spinal canal with removal of retropulsed fracture fragments	Patient recovered quickly and was discharged to inpatient behavior health.	Patient expressed suicidal ideation.
3	63	F	Off horse into a tree	grade 1-2 anterolisthesis of C3 upon C4, vertebral artery dissection	fusion of the anterior cervical spine with instrumentation of the C4 and posterior cervical fusion C3-C4	Patient recovered quickly and was able to be discharged home with family assistance	n/a
4	45	M	Motorcycle into a tree at 100MPH	Right parietal craniectomy defect, dural laceration	Emergent craniectomy	Discharge to skilled nursing facility	n/a
5	15	M	ATV into a tree, no helmet	Skull fx, SDH, T3-T4 fxs	Craniotomy and evacuation of SDH and EDH	Patient regained neuro function and d/c home.	n/a
6	17	M	Car struck tree avoiding deer at 50MPH	type III odontoid fracture w angulation and displacement	manipulation and confirmation of the proper halo-vest placement	Discharge home	n/a
7	64	F	Car struck tree	ruptured choroidal aneurism	Coiling of left anterior choroidal aneurism	Patient discharge to Helen Hayes rehab center.	n/a
8	44	M	Tree branch struck while hiking	T12, L1 fx	thoracolumbar fusion from T10-L2	Discharge to inpatient rehab	n/a
9	17	M	Car hit tree	Type II odontoid fracture and a C3 pedicle fracture	odontoid screw placement	Discharge home	n/a
10	30	F	Car hit tree	T12 vertebral body compression fracture with retropulsion	T10-L2 posterior spinal fusion	Patient improved significantly and was discharged home despite recommendation for inpatient.	n/a
11	31	M	Car hit a tree while intoxicated	C4/C5 fractures and small frontal left parenchymal bleed	Posterior cervical laminectomy and fusion of C3-C6.	Discharge to skilled nursing facility.	n/a
12	52	M	Fell out of a tree	Fx of temporal bones, skull base, and right orbit spinal fracture at the level of L1-L2	EVD placement	Patient expired	Progressive neurological decline from trauma.
13	17	F	Motorcycle hit tree	comminuted right temporal bone fracture as well as a comminuted right frontotemporal bone fracture, multiple facial fractures, nasal bone fracture, orbital wall fractures, bilateral sphenoid sinus fracture with extension into the right carotid canal, extra-axial hemorrhage to the right frontal and temporal region, scattered subarachnoid hemorrhages and epidural hematoma	EDH evacuation	Patient discharged to TBI rehab facility.	n/a
14	59	M	Car hit a tree while intoxicated	C1 fx	C1-C5 fusion	Discharge to an acute care rehab facility	Alcohol withdrawal, Pneumonia and ARDS secondary to tracheostomy
15	28	M	Fell out of a tree after skydiving accident	right subdural hematoma… right to left midline shift of 8 mm with herniation	Decompressive right hemicraniectomy	Discharge to acute TBI rehab facility.	Reactive thrombocytosis, required PEG/trach.
16	20	M	Cat hit a tree at high speed	type III odontoid fracture, C2 ligamentous injury, epidural hematoma from C1-C3, T3 burst fracture, left vertebral artery injury	Right hemicraniectomy and subsequent right cranioplasty	Discharge to Helen Hayes acute rehab. Returned for cranioplasty.	ARDS and respiratory failure requiring trach.
17	39	M	Fell out of tree and branch fell on him	depressed frontal skull fracture… large right frontal epidural hematoma with associated right frontal subarachnoid hemorrhage and subdural products	decompressive bifrontal craniectomy and skull base repair, subsequent cranioplasty and septorhinoplasty	The patient was discharged to Helen Hayes acute rehab facility. The patient returned for cranioplasty and was discharged home on POD#2.	Continued bleeding and pneumocephalus required no extra intervention.
18	68	F	Car hit tree	lumbar transverse spine fractures at L2 and L3 and unstable cervical C6-C7 spine fracture	placement of cervical traction tongs, C6-C7 ACDF, C6-C7 posterior instrumented fusion, bilateral C6 inferior facetectomies and foraminotomies, and a partial C6 laminectomy	Patient discharged to a subacute care facility	n/a
19	40	F	Tree fell on the car	C7-T1 spine fractures	C5-T1 posterior instrumented fusion with ORIF of the C7-T1 fracture, placement of 3 Pipeline Embolization Device stents	Discharge to an acute care facility.	Dissection of left ICA, treated by pipeline embolization.
20	46	F	Hit by a tree branch	right frontal skull fracture and traumatic intracranial hemorrhage	wound washout of the right open frontal skull fracture and cranioplasty with wound revision	Discharge to acute care rehab facility.	Persistent CSF rhinorrhea. The decision was made to place a lumbar drain, which resulted in a good resolution of the leak
21	35	M	Struck by branch he cut down	comminuted depressed right temporal bone fracture, Pneumocephalus, Extensive hemorrhagic contusive changes	elevation of skull fracture, irrigation, and debridement of the wound and removal of any foreign bodies	Discharge home	n/a

Illustrative cases

Case #1

**Figure 1:**
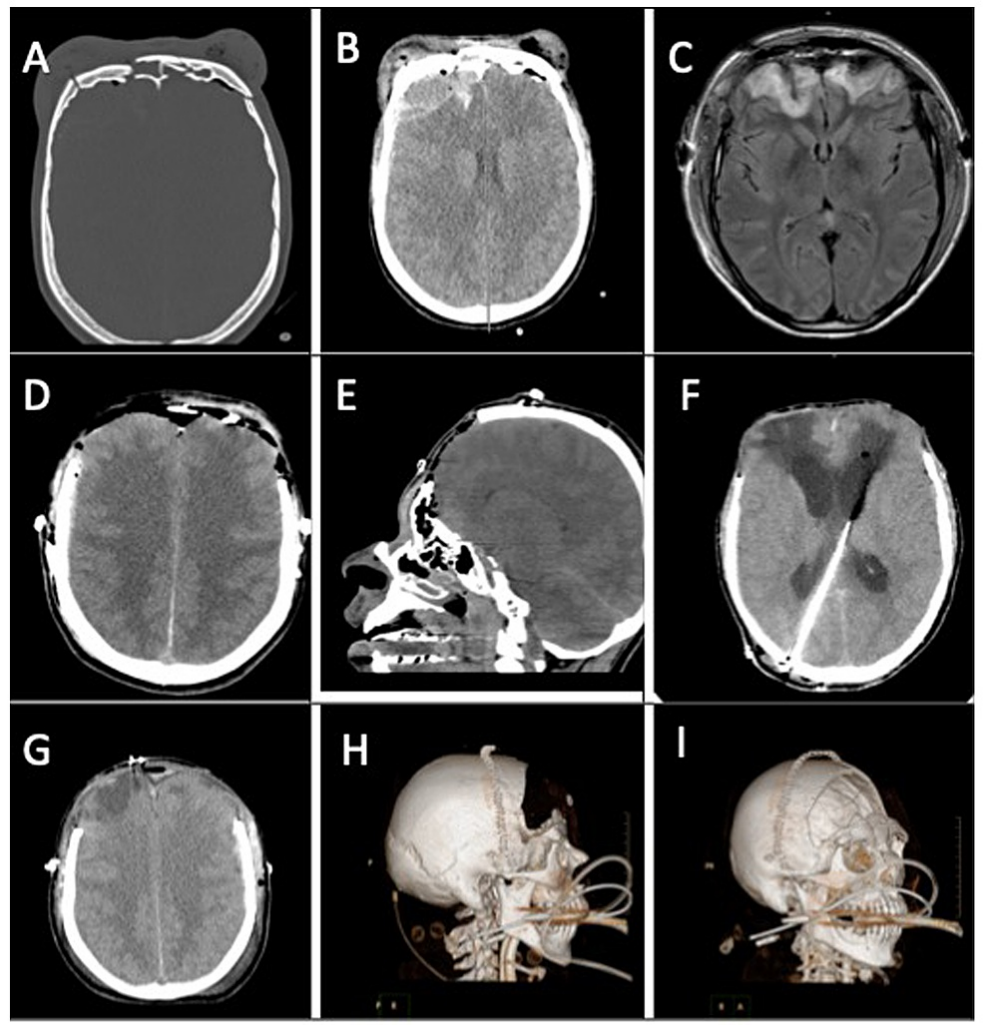
Neuroimaging findings of Case #1 A-B: CT of the head revealing extensive comminuted bilateral frontal bone fractures that violate the anterior and posterior walls of the frontal sinuses as well as a right frontal epidural hematoma, C: MRI of the brain revealing bilateral frontal lobe T2 FLAIR hyperintensities indicative of bifrontal contusions, D-E: Postoperative CT of the head following bifrontal decompressive craniectomy, F: Postoperative CT of the head following placement of a right occipital ventriculoperitoneal shunt, G-I: Postoperative CT of the head and 3-D image reconstruction following cranioplasty

Figure 1 shows the neuroimaging of case #1. This 39-year-old male landscaper was brought in by emergency medical services after sustaining a 16-feet fall from a tree while working. The patient’s initial exam in the trauma bay was consistent with a Glasgow coma scale (GCS) of nine (E1V2M6). However, the patient began to seize, requiring intubation for airway protection. Computed tomography (CT) of the head revealed a large right frontal epidural hematoma (EDH) with associated four mm right to left midline shift and scattered subarachnoid hemorrhage underlying depressed, comminuted frontal skull fracture, fractures through the posterior table of the frontal sinus, and extensive orbital wall fractures. Other injuries sustained included a comminuted right third rib fracture extending into the T2-T3 neural foramen and a Grade one blunt cerebrovascular injury (BCVI) of the left cervical internal carotid artery.

Due to the patient’s poor neurological exam and extensive open comminuted skull fractures, the patient was taken to the operating room for a decompressive bifrontal craniectomy. The patient was slow to progress from a neurological standpoint and was unable to be weaned from mechanical ventilation and required placement of a tracheostomy and percutaneous endoscopic gastrostomy (PEG) tube for enteral feeding access. Two months after his initial trauma, the patient developed post-traumatic hydrocephalus as evidenced by ventriculomegaly with transependymal flow as seen on imaging. A right-sided occipital ventriculoperitoneal shunt was placed prior to proceeding with cranioplasty with a custom-made Polyetheretherketone (PEEK) frontal implant, several days later. The patient’s postoperative course was uneventful, despite a single return to the operating room for washout and repair of a focal region of a scalp wound dehiscence. The patient was discharged to an acute rehab facility and has not had any other complications in the two years since surgery.

Case #2

**Figure 2:**
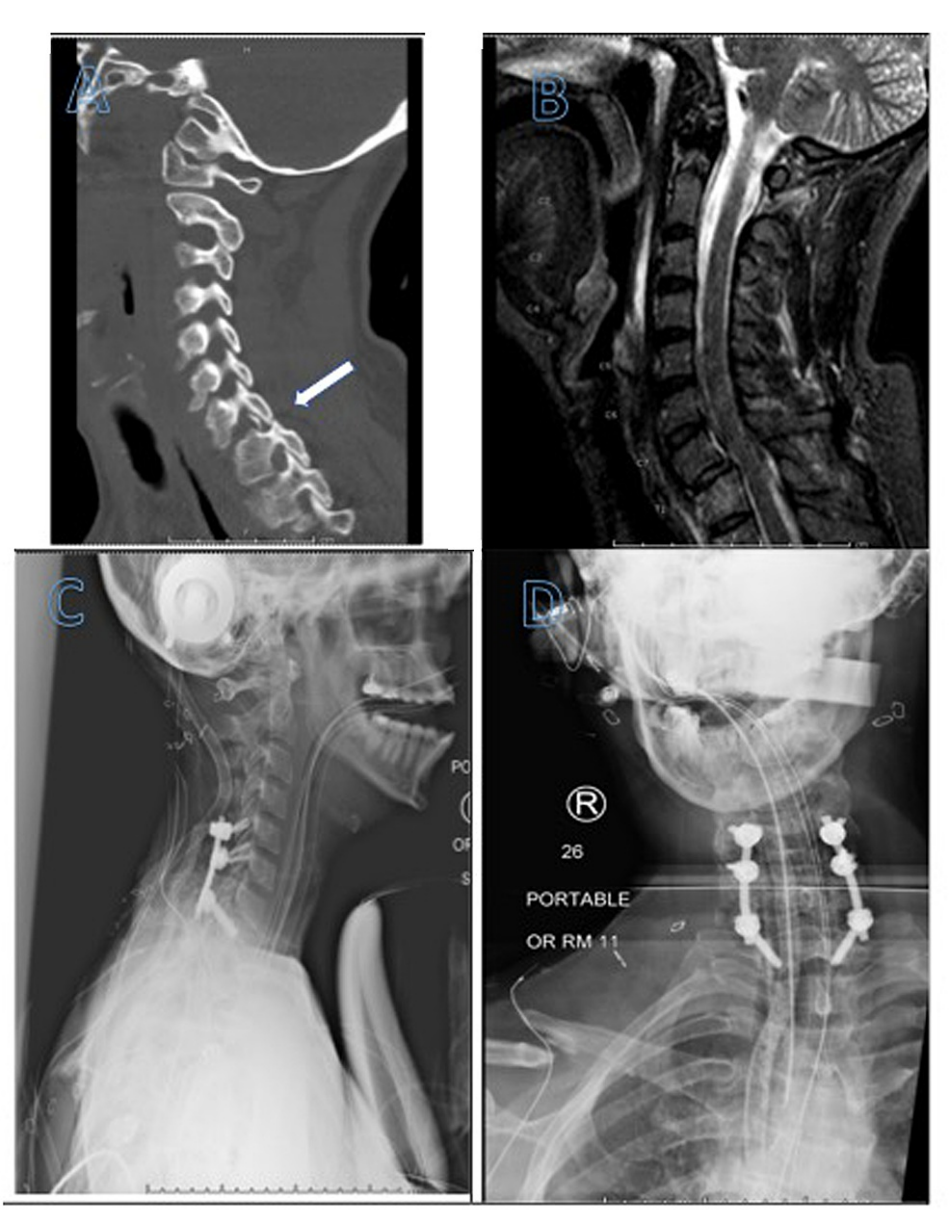
Neuroimaging findings of Case #2 A-B: Sagittal view computed tomography image and MRI of the cervical spine revealing a fracture-dislocation at C7-T1 with perched facets and 2-3 mm of anterolisthesis of C7 on T1, C-D: Anterior and lateral radiographs demonstrating postoperative reduction of the C7-T1 fracture and C5-T1 posterior instrumented fusion construct

Figure 2 shows the neuroimaging of case #2. A 40-year-old female was brought in by ambulance after a tree had fallen on top of her car. She was noted to have a right clavicular fracture and a fracture-dislocation at C7-T1 with perched and locked facets and 3 mm anterior subluxation of C7 over T1. CT of the spine revealed a fracture-dislocation at C7-T1 with perched and locked facets, as well as right superior articular process fracture of C7. CT of her head revealed trace traumatic subarachnoid hemorrhage in the left frontal convexity and a 4 mm left falcine subdural hematoma. Computed tomography angiography (CTA) of the neck revealed focal region of luminal irregularity of the left cervical internal carotid artery (ICA) just proximal to the skull base, consistent with Grade II blunt cerebrovascular injury (BCVI). On examination, the patient was found to be neurologically intact, without evidence of any significant weakness or evidence of myelopathy. The decision was made to take the patient to the operating room for C5-T1 posterior instrumentation and reduction of the subluxed C7-T1 segments. The patient tolerated the procedure well without complication. The patient was started on Aspirin 325 on postoperative day three for management of Grade II BCVI. Repeat CTA one week post-trauma, revealed further progression of the luminal narrowing of the left cervical ICA. The patient was taken to the angiography suite for diagnostic angiography which revealed focal region of >70% stenosis of the left distal cervical ICA extending into the petrous segment of the ICA and a secondary 13 mm traumatic pseudoaneurysm as well as a region of the right V1 segment vertebral artery dissection. A pipeline flow diverting stent was deployed across the left ICA dissection flap and pseudoaneurysm without complication. The patient was started on dual antiplatelet therapy (DAPT) with aspirin and clopidogrel and remained neurologically intact. The remainder of her hospital course was unremarkable, and the patient was discharged to an acute rehabilitation facility for further rehab.

Case #3

**Figure 3:**
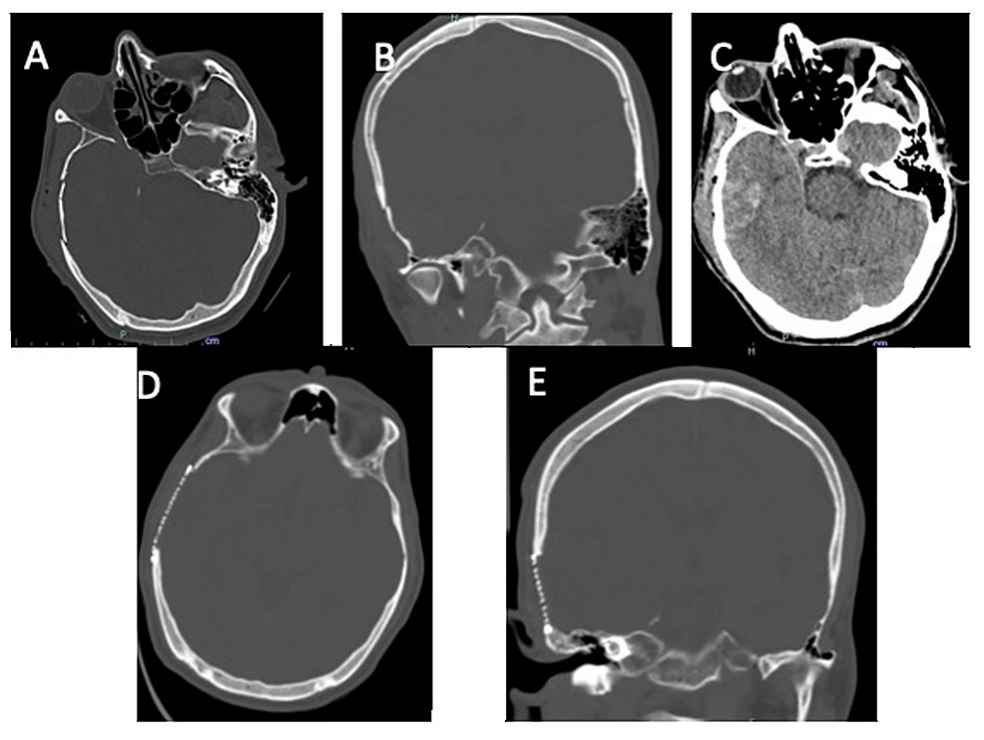
Neuroimaging findings of Case #3 A-B: Axial and coronal views on CT of the head revealing acute comminuted depressed right temporal bone fracture, C: Right temporal lobe contusion underlying the right temporal bone fracture, D-E: Postoperative CT of the head revealing titanium mesh cranioplasty following elevation and repair of the depressed temporal bone skull fracture

Figure 3 shows the neuroimaging of case #3. A 35-year-old male was cutting tree branches while suspended in a tree when a large branch fell and struck him on the right side of his head. The patient was not wearing a helmet and lost consciousness but did not fall from the tree as he was suspended by restraints. The patient was brought to the trauma bay by emergency medical services where he was noted to have a deep laceration over his right ear that probed down to the bone. CT of the head revealed a comminuted depressed temporal bone fracture underlying the open scalp defect with evidence of right temporal lobe contusions. The patient was taken to the operating room for washout and elevation of his skull fracture. The patient was maintained on intravenous antibiotics for a total of 14 days. On day 14 postoperatively, the patient was discharged home.

## Discussion

The high prevalence of neurologic injury that was seen resulting from tree-related trauma is concerning and therefore necessitates its reporting in the literature. The United States northeast contains the highest concentration of forested areas in the country, and therefore, individuals living in this area are at higher risk for suffering from tree-related trauma. While trees normally pose a slight risk to individuals, certain activities and behaviors increase the chance of sustaining such trauma from interactions with trees in the environment.

We found that serious injuries, many of which required neurosurgical intervention, occurred from working in a heavily forested area without proper safety protection. Between 2011 to 2015 Hakakian et al. noted that at their institution in New Jersey, and specifically in 2012 during Hurricane Sandy, 487 patients were admitted for tree-related trauma [4]. Of these patients, a larger percentage of patients with tree-related trauma were deceased compared with non-tree-related trauma (1.65% vs 0.53%, P = 0.001). Additionally, 20% of these patients were admitted to the intensive care unit, and 12 % were treated operatively. Furthermore, Hakakian et al. reported that tree-related trauma caused a larger average Injury Severity Score than non-tree-related trauma (9.5 vs 6.9, P < 0.005) [4].

Certain factors were found to compound the danger of living in an area that is heavily forested. Of the injuries discussed in this paper majority of them could have been prevented or the injury severity reduced if proper safety precautions were used. Additionally, four injuries were sustained by intoxicated patients, with three of these patients operating motor vehicles prior to their vehicle colliding with a tree. Adding to this, a study published by Liu et al. found that motorcyclists involved in a motor vehicle crash while intoxicated had an increased likelihood of GCS ≤ 8 (20.3%) than those not intoxicated (16.2%) (p=0.004), and a lower mean GCS score overall (12.1±3.9 vs 12.9 ±3.6, N = 601 vs 829, P = 0.003) [5]. Hakakian et al. noted that in terms of trauma related to motor vehicle collisions (MVC), a higher injury severity score was observed in MVCs involving trees, than in MVCs not involving trees [4]. Additionally, another study evaluating 287 motor vehicle accidents, noted that 19% were tree collisions. Interestingly, the authors noted that 36/54 tree collisions occurred on straight roadways and that the majority of patients sustained more than one injury [6].

Compliance with certain safety recommendations, including driving while fully alert and responsive, as well as following all posted speed limits, can help prevent some of the injuries presented. In addition, the continued application of other motor vehicle safety precautions, such as wearing seatbelts, can further increase safety and reduce the prevalence of all motor vehicle-related injuries, including those involving trees.

A paper by Gagnon et al. discussed the injuries seen from falling trees and from improper tree clearance following Hurricane Isabel in Virginia in 2005 [7]. The authors noted that most of the injuries occurred as a result of individuals trying to clear their own fallen trees or trees in their neighborhood [7]. The authors also found that of all tree-related trauma patients, only one was a professional tree cutter who wore protective equipment [7]. A similar study conducted by the Center for Disease Control (CDC) following Hurricane Charley in 2004 found that the majority of deaths during Hurricane Charley involved blunt trauma caused by injuries from falling trees, flying debris, and destroyed physical structures [8].

While not reported in the current case series, an additional risk of tree-related trauma is an injury during snow sports such as skiing and snowboarding. It has been reported that head injuries comprise up to 15% of snow sports injuries [9]. In fact, in one study evaluating 350 snow sports injuries admitted to a trauma center, the skier-tree collision had a mortality rate of 7.2%, which was the highest of all injury mechanisms evaluated. For patients who did survive, GCS scores following skier-tree collisions were more often in the range of three to eight, highlighting the devastating nature of these injuries. It must also be noted that this study reported that only one patient was wearing a helmet at the time of injury, and this patient’s injuries were limited to a concussion [10]. In another study of 107 patients admitted to a level one trauma center for evaluation following snow sport injury, 33% sustained injuries following skier-tree collisions. Moreover, injuries such as skull fracture and head injury were more commonly reported in the tree collision cohort as compared to the non-tree-collision cohort which highlights the need for greater awareness of these injuries, especially within the neurosurgical community [11].

Proper medical treatment of tree-related neurotrauma is imperative. Rapid triage and examination are essential to evaluate the severity of the injury and to provide severely injured patients with effective treatment. Early imaging with CT or X-ray is critical to determine if emergent neurosurgical intervention in the OR is needed, or if a watch and wait approach can be employed. Finally, determination of the need for emergent surgical intervention is imperative for the survival and outcome of the patient.

## Conclusions

The present study was undertaken to more clearly elucidate the types of injuries sustained due to tree-related trauma and to describe the relationship between mechanism and neurological severity. An analysis of our series and a review of the literature demonstrates that serious head injuries secondary to tree-related trauma cannot be overlooked. Many of these injuries can be prevented or reduced in severity by adhering to safety recommendations in place. Even with such safety recommendations, tree-related trauma will continue to be seen, and rapid and effective treatment strategies must be employed to ensure the best possible long-term outcome.

## References

[1] Nowak DJ, Greenfield EJ (2012). Tree and impervious cover in the United States. Landscape and Urban Planning.

[2] Hamilton K, Rocque B, Brooks N (2017). Spine and spinal cord injuries after falls from tree stands during the Wisconsin deer hunting season. WMJ.

[3] Walsh RA, Ryan L (2017). Hospital admissions in the Hunter Region from trees and other falling objects, 2008-2012. Aust N Z J Public Health.

[4] Hakakian D, Del Rosario AG, Bogdanovski DA (2018). Analysis of injury patterns due to tree-related trauma. Am Surg.

[5] Liu HT, Liang CC, Rau CS, Hsu SY, Hsieh CH (2015). Alcohol-related hospitalizations of adult motorcycle riders. World J Emerg Surg.

[6] Matthes G, Schmucker U, Schindel M, Siebenhühner S, Ekkernkamp A, Seifert J (2007). Tree collisions in road traffic accidents - mechanism and pattern of injury (Article in German). Zentralbl Chir.

[7] Gagnon EB, Aboutanos MB, Malhotra AK, Dompkowski D, Duane TM, Ivatury RR (2005). In the wake of Hurricane Isabel: a prospective study of postevent trauma and injury control strategies. Am Surg.

[8] Centers for Disease C, Prevention Prevention (2004). Preliminary medical examiner reports of mortality associated with Hurricane Charley--Florida, 2004. MMWR Morb Mortal Wkly Rep.

[9] Levy AS, Smith RH (2000). Neurologic injuries in skiers and snowboarders. Semin Neurol.

[10] Levy AS, Hawkes AP, Hemminger LM, Knight S (2002). An analysis of head injuries among skiers and snowboarders. J Trauma.

[11] Friermood TG, Messner DG, Brugman JL, Brennan R (1994). Save the trees: a comparative review of skier-tree collisions. J Orthop Trauma.

